# Coronary sinus atrial septal defects in adults over the past 20 years at new Tokyo hospital: case series

**DOI:** 10.1186/s13019-021-01522-x

**Published:** 2021-05-29

**Authors:** Haruhiko Sugimori, Tatsuya Nakao, Yuki Ikegaya, Daisuke Iwahashi, Shoichi Tsuda, Nao Kume, Hirokazu Onishi, Sunao Nakamura

**Affiliations:** New Tokyo Hospital, 1271 Wanagaya, Matsudo, Chiba, 270-2232 Japan

**Keywords:** Case series, Coronary sinus atrial septal defect, Unroofed coronary sinus syndrome, Poor prognosis, Renal dialysis, Adult congenital heart disease

## Abstract

**Background:**

An isolated coronary sinus (CS) atrial septal defect (ASD) is defined as a CS unroofed in the terminal portion without a persistent left superior vena cava or other anomalies. This defect is rare and part of the wide spectrum of unroofed CS syndrome (URCS). Recently, several reports have described this finding. The database of New Tokyo Hospital was searched to determine the incidence of this defect. Additionally, to raise awareness of this condition, the findings from five patients with CS ASD who underwent surgical repair at New Tokyo Hospital are discussed.

**Case presentation:**

The patients were three women and two men with an age range of 63–77 years. All patients underwent transthoracic echocardiography and computed tomography, and one underwent magnetic resonance imaging. In two patients, the defect was found unexpectedly intraoperatively; left-to-right shunting was apparent in the other three patients preoperatively. The pulmonary-to-systemic blood flow ratio ranged from 1.42 to 3.1 following cardiac catheterization, and oxygen saturation step-up was seen on the right side of the heart. Valvular regurgitation was seen in 4/5 patients with different combinations and degrees of mitral, tricuspid, and aortic valve involvement. Right atrial and ventricular dilation were seen in 4/5 patients; three patients had left atrial dilation. Three patients experienced atrial fibrillation, and one of these also experienced paroxysmal ventricular contractions. All patients underwent surgical repair, and some underwent multiple procedures. One patient who had previously undergone kidney transplantation died approximately 1 year postoperatively; the remaining four patients are currently experiencing good activities of daily living without symptoms.

**Conclusions:**

CS ASD (Kirklin and Barratt–Boyes type IV URCS) comprised 1.3% of adult congenital heart surgeries and 0.07% of adult open-heart surgeries at New Tokyo Hospital from 1999 to 2019. At New Tokyo Hospital, cardiac surgery is performed mainly for patients with acquired cardiac disease, and CS ASD is rare. Early diagnosis is important, as well as early surgical repair in symptomatic patients, especially those with blood access shunts, which may overload the heart. The case of a poor prognosis in this series is noteworthy, as similar cases have not been reported previously.

**Supplementary Information:**

The online version contains supplementary material available at 10.1186/s13019-021-01522-x.

## Background

Unroofed coronary sinus syndrome (URCS) is a rare congenital heart disease [1, 2]. Coronary sinuses (CSs) unroofed in the terminal portion (Kirklin and Barratt-Boyes type IV) [3] without a persistent left superior vena cava (PLSVC) or other anomalies are classified as a type of atrial septal defect (ASD) (“isolated CS ASD”), which comprises less than 1% of all ASDs in the literature [3–5]. Several recent reports have discussed this anomaly to raise awareness of its existence [6–9]. Therefore, this investigation of the medical database of New Tokyo Hospital was performed, and experience with the repair of CS ASD at this institution was examined via a retrospective review of the hospital’s surgical records from the past 20 years, which confirmed the existence of and survival outcomes associated with this anomaly.

In general, the prognosis after repair is considered to be favorable, with an uneventful course. Reviewing past reports did not reveal any patients with a poor prognosis after repair of the same type of anomaly. Although some surgeons specializing in congenital heart disease may have the impression that CS ASD is not a concern, especially in hospitals performing surgery mainly for adult patients with acquired heart disease, CS ASD is very rare. The aim of this investigation was to discuss the epidemiology of and clinically important points related to this anomaly by reviewing the surgeries performed for congenital heart disease at New Tokyo Hospital, where surgeries are performed mainly for adult acquired heart disease.

## Case presentation

All included patients underwent surgical CS ASD repair between January 1999 and December 2019. Upon review of the hospital’s surgical records from the past 20 years, five cases (as described below chronologically) of CS ASD were identified, and the survival of the patients was confirmed. Their background characteristics are shown in Table 1.

**Table 1:** Background characteristics of patients with coronary sinus atrial septal defect

Characteristic	Case 1	Case 2	Case 3	Case 4	Case 5
Height (cm)	171	159	147	168	158
Weight (kg)	67	53	42	66	40
BSA (m2)	1.74	1.50	1.34	1.75	1.36
Cardiac rhythm	AF	AF	NSR	NSR	AF
HT	+	+	–	+	+
DM	–	–	–	–	–
DL	–	–	+	+	–
OMI	–	–	–	–	–
COPD	+	+	–	–	–
CKD	–	–	–	–	HD since 1982
Previous cardiovascular surgery	–	–	–	–	Blood access shunt construction
Previous PCI	–	–	–	–	–
Others	–	RFCA Cesarean sec. Appendectomy	–	–	Renal transplantation from a deceased donor Pancytopenia

*BSA* body surface area, *NSR* normal sinus rhythm, *AF* atrial fibrillation*, HT* hypertension, *DM* diabetes mellitus, *DL* dyslipidemia, *OMI* old myocardial infarction, *COPD* chronic obstructive pulmonary disease, *CKD* chronic kidney disease, *HD* hemodialysis, *PCI* percutaneous coronary intervention, *RFCA* radiofrequency catheter ablation, *cesarean sec*. cesarean section

Surgical cases of adult congenital heart disease and the number of open-heart surgeries were also investigated. Cases of off-pump coronary artery bypass grafting were included as open-heart surgeries, and patent foramen ovale was excluded from the investigation as a congenital heart disease (Supplemental Table 1).

This research was approved by the hospital’s ethics committee and the president of the hospital. The ethics committee waived the requirement to obtain informed consent for the use of patients’ data except for in the five cases included in this retrospective study.

### Case 1

A 63-year-old man experienced paroxysmal atrial fibrillation (AF) from the age of 33 years. Ostium secundum ASD was identified at age 42 at another hospital. He had undergone follow-up for 11 years at another hospital, and when chest X-ray (CXR) showed cardiomegaly with a cardiothoracic ratio (CTR) of 70%, he was referred to New Tokyo Hospital. Transthoracic echocardiography (TTE) showed significant dilation of the right atrium (RA), right ventricle (RV), and left atrium (LA), as shown in Table 2, an ASD measuring 30 mm, and left-to-right intracardiac shunting. Moreover, TTE showed moderate mitral regurgitation (MR) due to prolapse of the anterior mitral leaflet and severe tricuspid regurgitation (TR) with a TR pressure gradient of 54 mmHg (Table 2). Cardiac catheterization revealed a Qp/Qs of 3.1 and a pulmonary-to-systemic pressure ratio (Pp/Ps) of 0.5 (Tables 3 and 4). He was admitted for surgery on 2 November 2005.

**Table 2:** Preoperative transthoracic echocardiography data

Measurement	Case 1	Case 2	Case 3	Case 4	Case 5
BSA (m2)	1.74	1.50	1.34	1.75	1.36
CS orifice (mm or mm × mm	–	23	21 × 19	24 × 18	28 × 27
Defect size (mm)	–	–	16	–	13
Qp/Qs	4.2	–	2.1	2.0	2.7
RA 4ch (mm × mm)	70 × 105	46 × 69	42 × 56	43 × 61	41 × 73
RV 4ch (mm × mm)	57 × 80	–	39 × 65	47 × 92	31 × 72
TR	Severe	Mild	Moderate to severe	Trivial	Moderate
TRPG (mmHg)	54	22	41.3	19.0	27.4
LA 4ch (mm × mm)	53 × 97	48 × 70	42 × 62	43 × 64	48 × 83
LVIVST (mm)	10	9.0	8.4	10.0	12.0
PWT (mm)	10	8.5	8.1	9.2	12.0
Dd (mm)	44	44	38	47	40
Ds (mm)	29	33	25	29	26
EF (%)	64.0	49.2	65.6	68.0	63.3
MR	Moderate	Mild	Trivial	Trivial	Mild to moderate
AR	–	Moderate	Trivial	–	Trivial
PR	Trivial	Trivial	Mild	Trivial	Mild

*BSA* body surface area, *CS* coronary sinus, *Qp/Qs* pulmonary-to-systemic blood flow ratio, *RA 4ch* right atrial diameter on four-chamber view, *RV 4ch* right ventricular diameter on four-chamber view, *TR* tricuspid regurgitation, *TRPG* tricuspid regurgitant pressure gradient, *LVIVST* left ventricular interventricular septal thickness, *PWT* left ventricular posterior wall thickness, *Dd* left ventricular end-diastolic dimension, *Ds* left ventricular end-systolic dimension, *EF* left ventricular ejection fraction, *MR* mitral regurgitation, *AR* aortic regurgitation, *PR* pulmonary regurgitation

**Table 3:** Cardiac catheter flow study and pressure study results

	Qp/Qs	L–R shunt ratio (%)	R − L shunt ratio (%)	PAP s/m (mmHg)	PCWP (mmHg)	C.I. (L/min/m^2^)
Case 1	3.1	71.4	12.0	78/36	15	4.96
Case 4	1.42	35.4	8.4	19/8	1	5.32
Case 5	1.82	45.0	0.0	33/21	11	4.17

*Qp/Qs* pulmonary-to-systemic blood flow ratio, *L to R* left to right, *R to L* right to left, *PAP s/m* pulmonary artery pressure systolic/mean, *PCWP* mean pulmonary capillary wedge pressure, *C.I.* cardiac output index

**Table 4:** Cardiac catheter oxygen saturation study results

(%)	SVC	RA			RV			PA	PCW	Ao
		High	Middle	Low	Inflow	Apex	Outflow			
Case 1	71.1	89.4	88.0	89.0	90.3	89.3	89.5	90.1	97.8	94.9
Case 4	69.1	72.5	76.7	78.2	78.9	78.3	79.4	78.6	97.8	95.4
Case 5	71.1	70.8	71.6	78.8		84.2	82.5	81.4	95.0	95.0

*SVC* superior vena cava, *RA* right atrium, *RV* right ventricle, *PA* pulmonary artery, *PCW* pulmonary capillary wedge, *Ao* aorta

### Case 2

A 71-year-old woman had undergone regular follow-up to monitor AF. She underwent radiofrequency catheter ablation (RFCA) for frequent paroxysmal ventricular contractions (PVCs) focused on the right ventricular outflow tract (RVOT) in April 2011. Subsequently, she developed dyspnea with New York Heart Association (NYHA) class II heart disease, and she was referred to New Tokyo Hospital for aortic regurgitation (AR) progression in October 2012. Electrocardiography (ECG) showed AF but a stable heart rate of approximately 70 bpm. CXR showed cardiomegaly, with a CTR of 60%, and TTE showed moderate AR, mild-to-moderate MR, and definite dilation of the RA and LA. Although the TTE findings indicated CS dilation measuring 23 mm, no intracardiac shunt was identified preoperatively (Table 2). She was admitted for surgery on 5 January 2013.

### Case 3

**Fig. 1:**
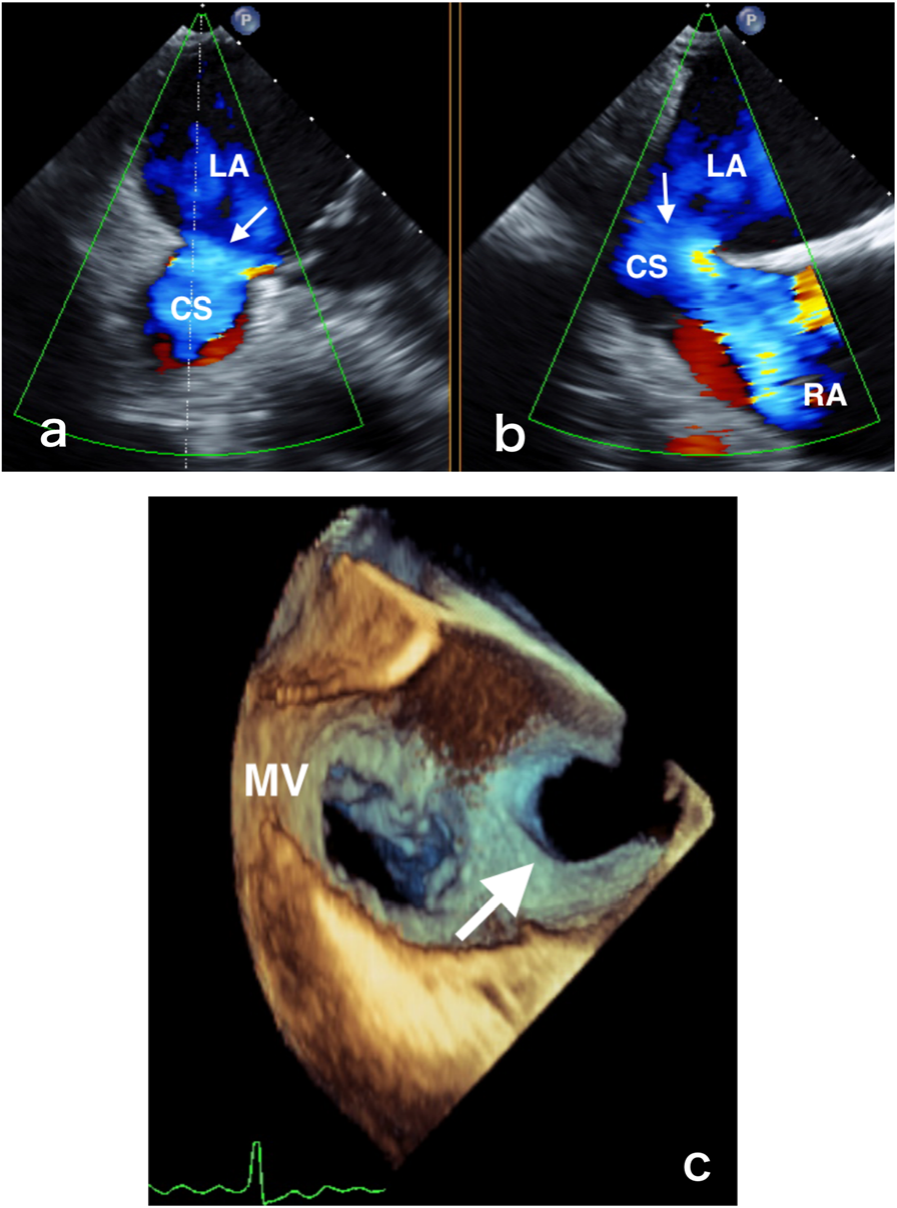
Echocardiographic findings in cases 3 and 5. **a**, **b** Dynamic left-to-right shunting visible through the defect in case 3. **c** Three-dimensional transesophageal echocardiography in case 5 clearly showing a defect (white arrow) near the posterolateral commissure of the mitral valve. *CS* coronary sinus, *LA* left atrium, *MV* mitral valve, *RA* right atrium

A 77-year-old woman experienced discomfort during calm physical exercise (yoga) for 2 years. Subsequently, she experienced episodes of presyncope. TTE and CT revealed an unroofed CS. She was referred to New Tokyo Hospital to confirm indications for surgical repair. CXR showed no significant signs of heart failure. Her heart rate was stable, with a normal sinus rhythm. TTE showed a large defect between the LA and the CS, measuring 16 mm in width. The Qp/Qs was estimated at 2.1, and severe TR was observed. The RA and RV were definitely dilated (Table 2). Transesophageal echocardiography (TEE) (iE33, Philips Medical Systems, Bothell, WA, USA) revealed a defect in the roof of the CS measuring 25.3 mm × 15.6 mm. The defect was located 8.9 mm from the CS orifice and 3.0 mm from the mitral valve annulus (Table 5), with clear left-to-right dynamic shunting through the defect (Fig. 1a and b). She was admitted for surgery on 14 March 2016.

**Table 5:** Preoperative transesophageal echocardiography findings

Measurement	Case 3	Case 4	Case 5
Defect diameter max x min [mm]	25.3 × 15.6	29.2 × 19.9	18.5 × 12.3
Distance from CS orifice [mm]	8.9	4.0	4.5
Distance from MV annulus [mm]	3.0	4.0	5.0

*CS* coronary sinus, *MV* mitral valve

### Case 4

**Fig. 2:**
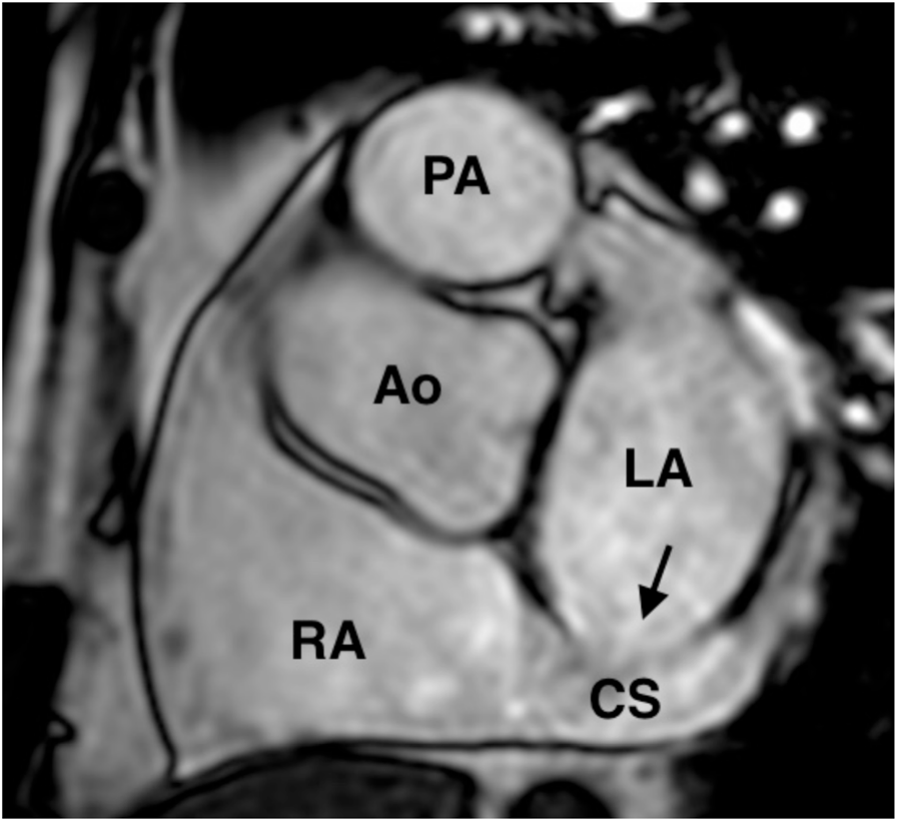
Magnetic resonance imaging in case 4. Magnetic resonance imaging (sagittal view) in case 4 showing the morphology of the patient’s congenital heart disease. We found no persistent left superior vena cava or other congenital cardiovascular anomalies. The arrow indicates the coronary sinus defect. *Ao* aorta, *CS* coronary sinus, *LA* left atrium, *PA* pulmonary artery, *RA* right atrium

A 65-year-old man was referred to New Tokyo Hospital with NYHA class II dyspnea. CXR showed an almost normal cardiac size, with a 51% CTR and no other signs of congestion. His heart rate was approximately 70 bpm with a sinus rhythm during ECG. TTE showed a dilated CS orifice measuring 24 mm × 18 mm, and shunt flow was visible at almost the same point, which suggested blood flow from the LA through the unroofed CS to the RA. The Qp/Qs was 2.0, and the RA and RV were definitely dilated (Table 2). TEE clearly showed a defect in the roof of the CS measuring 29.2 mm × 19.9 mm, located 4.0 mm from the CS orifice and 4.0 mm from the mitral valve annulus (Table 5). Magnetic resonance imaging (MRI, Achieva 3.0 T, Philips, Inc., Best, The Netherlands) confirmed this congenital disease, with no PLSVC or other congenital cardiovascular anomalies (Fig. 2). Cardiac catheterization resulted in a Qp/Qs of 1.42 and a Pp/Ps of 0.10 (Table 4). He was admitted for surgery on 23 July 2016.

### Case 5

**Fig. 3:**
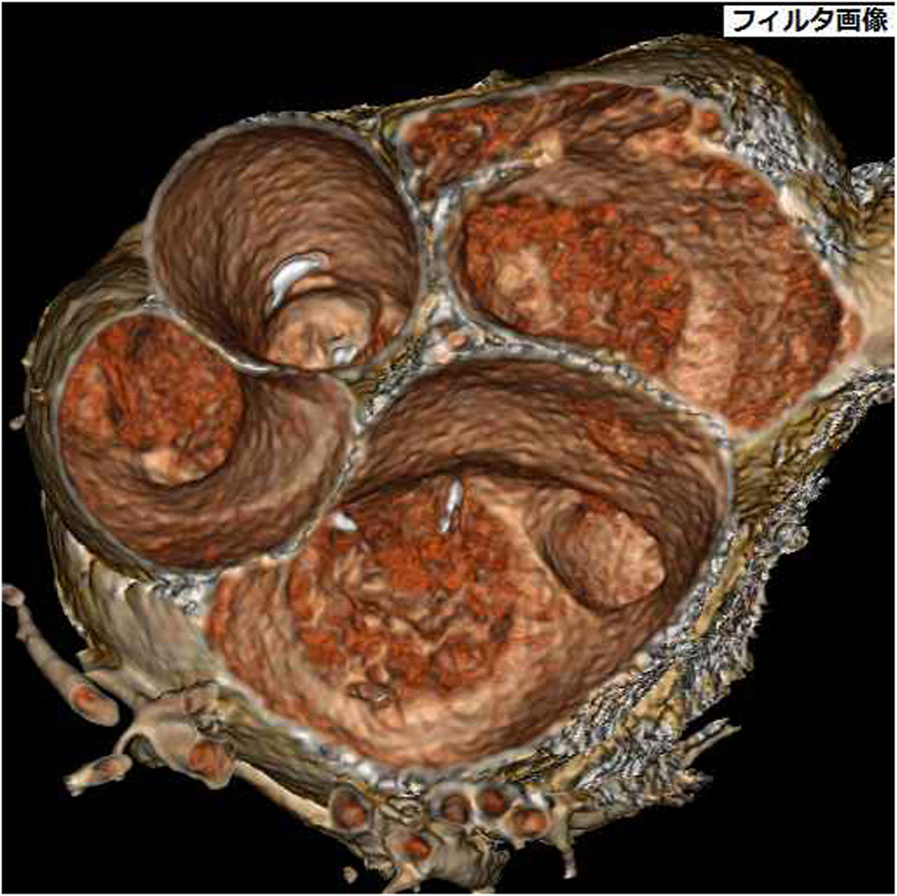
Sixty-four-row multidetector computed tomography in case 5. Sixty-four-row multidetector computed tomography in case 5 showing the morphology of the patient’s congenital heart disease. The arrow indicates the coronary sinus defect. *Ao* aorta, *MV* mitral valve, *PA* pulmonary artery, *TV* tricuspid valve

A 74-year-old woman was referred to New Tokyo Hospital with complaints of palpitations and dyspnea. She had started renal dialysis when in her 40s because of chronic glomerulonephritis, and at 46 years of age, she underwent renal transplantation from a deceased donor. However, she subsequently required constant renal dialysis. After transplantation, she began taking cyclosporine and underwent medical follow-up after the age of 63 years to monitor pancytopenia. On admission for heart surgery, she was taking 100 mg of cyclosporine once per day, and fortunately, her blood cell counts were in the normal ranges. CXR showed cardiomegaly, with a CTR of 59% and pulmonary congestion but no pulmonary effusion. Her cardiac rhythm showed AF, and her heart rate was approximately 70 bpm on ECG. TTE showed a dilated CS orifice measuring 28 mm × 27 mm and a 13-mm-wide defect on the roof of the CS. The Qp/Qs was estimated at 2.7, and moderate regurgitation in both the mitral and tricuspid valves was detected. The RA, RV, and LA were definitely dilated, and the left ventricle was mildly hypertrophic but had a normal ejection fraction and mild-to-moderate MR (Table 2). TEE clearly showed an approximately oval-shaped defect on the roof of the CS, which was measured to be 18.5 mm × 12.3 mm and located 4.5 mm from the CS orifice and 5.0 mm from the mitral valve annulus (Fig. 1c, and Table 5). Multidetector-row CT (SOMATOM Definition AS+; Siemens Medical Systems, Forchheim, Germany) confirmed this congenital disease (Fig. 3). No PLSVC or other congenital cardiovascular anomalies were identified. Cardiac catheterization revealed a Qp/Qs of 1.82 and a Pp/Ps of 0.3 (Table 3). She was admitted for surgery on 1 October 2016.

### Surgical procedure

On 7 November 2005, the patient in case 1 underwent secundum ASD patch closure with autologous pericardium, mitral annuloplasty (MAP), tricuspid annuloplasty (TAP), a biatrial maze procedure, and RA plication. Cardiopulmonary bypass was commenced by ascending aortic cannulation near the aortic arch and both superior vena cava (SVC) and inferior vena cava (IVC) cannulation. Aortic cross-clamping (AXC) was performed with antegrade cardioplegia (CP) through the aortic root. Under cardiac arrest, the secundum ASD was found to measure 30 mm × 40 mm, and an unroofed CS to the LA was identified. A retrograde CP cannula was inserted from the CS directly. After opening the right side of the LA, the mitral valve could be observed. Dilation of the mitral annulus, mild myxomatous degeneration on the leaflets, and a relatively normal subvalvular apparatus were observed. Maze cryoablation, MAP with a 30-mm ring, and TAP with a 36-mm band were performed. Finally, the secundum ASD was closed with an autologous pericardial patch using 5–0 polypropylene (ppp) running sutures. The CS ASD was also closed by direct suturing or the application of an autologous pericardial patch from the left atrial side.

On 15 January 2013, the patient in case 2 underwent aortic valve replacement with a 25-mm bioprosthesis, mitral valve repair (plasty) (MVP) with anterior commissurotomy, anterior mitral leaflet thinning by peeling of the thickened intima [10], MAP with a 28-mm ring, TAP with a 30-mm band, the biatrial maze procedure, and RA plication. Cardiopulmonary bypass was established by ascending aortic cannulation and both SVC and IVC cannulation. AXC and antegrade CP were performed. Under cardiac arrest, after opening the RA, a 10-mm communication was found between the LA and CS when the retrograde CP cannula was inserted from the CS directly. CS ASD was diagnosed, and closure was performed by direct suturing from the left atrial side through the opening of the right side of the LA.

In 2016, surgery for the repair of preoperatively diagnosed CS ASD was performed for the patient in case 3 on 16 March, that in case 4 on 25 July, and that in case 5 on 4 October, described below.

**Fig. 4:**
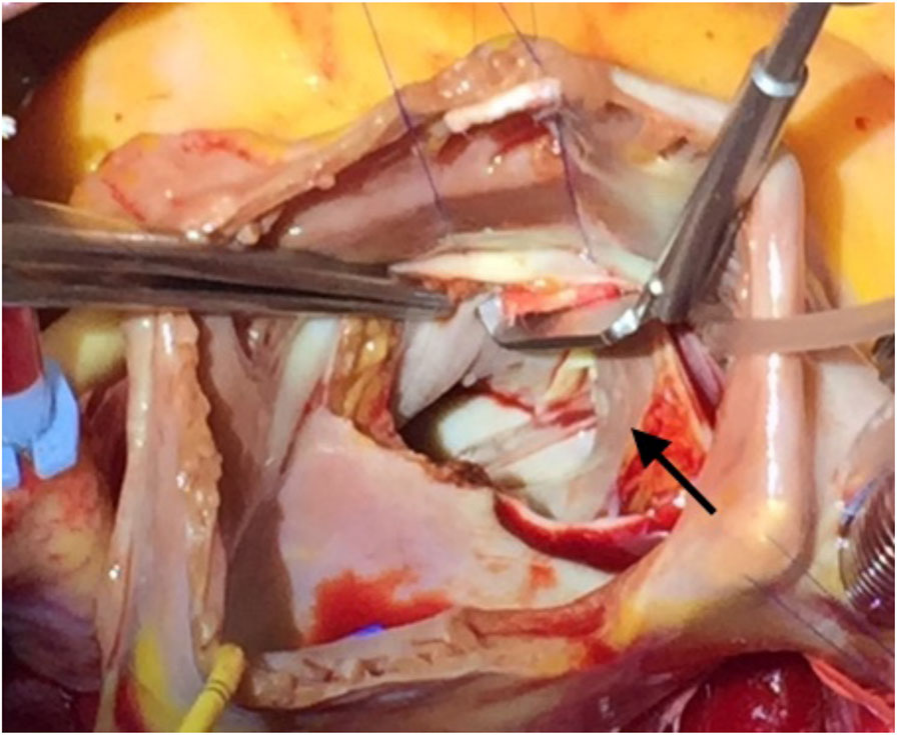
Coronary sinus defect in case 3. We confirmed communication between both atria through the defect (arrow) by inserting a catheter through the coronary sinus orifice. The image is a surgical photograph from case 3

Under general anesthesia and with a median sternotomy, cardiopulmonary bypass was performed between the ascending aorta and both vena cavae. Myocardial protection was achieved with cold-blood CP through the aortic root. After AXC and cardioplegic arrest, the RA was opened. With a trans-interatrial septal approach, the septum was retracted to clearly observe the mitral valve, and the CS defect was identified from the left atrial side. The defect was observed near the mitral posterior commissure (Fig. 4). The upper limbus was fibrous tissue, and the lower limbus was the muscular line, as seen under guidance from the retrograde cardioplegia cannula in the CS. The defect was closed using a fresh autologous pericardial patch (3 cm × 2.5 cm) with 5–0 ppp continuous sutures. To open the CS, a Nelaton tube was placed in the CS when the deep side of the patch edge was sutured.

In case 3, TAP with a 26-mm band was added. In case 5, MAP was performed with a 28-mm ring, along with valvuloplasty on both the P1–P2 and P2–P3 indentations, TAP with a 30-mm band, and a left atrial maze procedure (Table 6).

**Table 6:** Operative and postoperative results in five cases of coronary sinus atrial septal defect

	Case 1	Case 2	Case 3	Case 4	Case 5
URCS type	IV	IV	IV	IV	IV
PLSVC	–	–	–	–	–
AXC time (min)	114	223	99	95	147
Repeat AXC time (min)	–	–	47	48	–
CPB time (min)	199	258	207	180	183
Concomitant surgeries	MAP, TAP, maze, RA plication	AVR, MVP, TAP, maze, RA plication	TAP	–	MVP, TAP, maze, LAAA
Extubation (POD)	0	1	1	1	1
ICU stay (days)	1	2	5	2	10
Hospital stay (days)	13	25	22	15	28
Early complications	–	–	–	–	–
Late complications	HD since 2016	–	–	–	CSM Disuse syndrome Anemia Hypoproteinemia Hypotension
Survival	Alive	Alive	Alive	Alive	Dead

The morphology of URCS was classified as reported by Kirklin and Barratt-Boyes, as follows: type I, completely unroofed with PLSVC; type II, completely unroofed without PLSVC; type III, partially unroofed midportion; and type IV, partially unroofed terminal portion [3]

*URCS* unroofed coronary sinus syndrome, *PLSVC* persistent left superior vena cava, *AXC* aortic cross-clamping, *re-AXC* repeat aortic cross-clamping, *CPB* cardiopulmonary bypass, *TAP* tricuspid annuloplasty, *MVP* mitral valve repair (plasty), *LAAA* left atrial appendage amputation, *AVR* aortic valve replacement, *RA* right atrium, *MAP* mitral annuloplasty, *POD* postoperative day, *ICU* intensive care unit, *HD* hemodialysis, *CSM* cervical spondylotic myelopathy

In cases 3 and 4, after aortic declamping, TEE showed a residual shunt at the site of the patch intraoperatively. Therefore, secondary AXC was immediately performed, the cardioplegic arrest was reversed, the site of patch closure was carefully observed, and a leakage site was found near the mitral posterior commissure. At the site, a small fold was present in the running suture line, which was corrected with additional sutures, closing the leak. The precise AXC duration is shown in Table 5.

### Outcomes

These patients had uneventful postoperative courses and were discharged approximately 2–4 weeks after the operation (Table 6). All patients except for the patient in case 5 are currently experiencing very good activities of daily living without symptoms. Although the patient in case 1 has been on renal dialysis due to renal sclerosis since April 2016, i.e., 5 months and 10 years after the operation, he has been living in a very good condition for 15 years after the repair. Unfortunately, the patient in case 5 developed sustained chronic AF, anemia, hypoproteinemia, progression of disuse syndrome from cervical spondylotic myelopathy, and difficulties during renal dialysis due to hypotension despite good ventricular contraction. The patient died approximately 1 year after the operation at another hospital (Table 6).

From January 1999 to December 2019, five patients with CS ASD were identified: one in 2005, one in 2013 and three in 2016. In the past 20 years, 371 adult congenital cardiac surgeries (median age: 61 years, range: 13–88 years; mean age ± standard deviation: 56 ± 17 years; males: 210; females: 161) and 7314 open-heart surgeries were performed (Supplemental Table 1). CS ASD comprised 1.3% of adult congenital heart surgeries and 0.07% of adult open-heart surgeries (Supplemental Table 1). In surgery for adult congenital heart disease, procedures for acquired cardiovascular diseases, such as valvular surgeries, coronary artery bypass grafting, and aortic surgeries, are usually performed concomitantly.

## Discussion and conclusions

There were five patients who underwent CS ASD repair at New Tokyo Hospital from 1999 to 2019. In the last 20 years at the hospital, where cardiac surgeons perform operations mainly for adult acquired heart disease, CS ASD comprised 0.07% of open-heart surgeries, so this condition is a very rare anomaly at this hospital. These were also cases of the simplest type of URCS; therefore, in general, the prognosis after repair is considered to be favorable, with an uneventful course. Unfortunately, the patient in case 5 died approximately 1 year after the operation. Reviewing past reports revealed no patients with a poor prognosis after CS ASD repair (Kirklin and Barratt-Boyes type IV URCS) without other complex congenital anomalies, i.e., case 5 provided novel insights. In case 5, volume overload of the cardiac chambers resulted in AF, annular dilatation of the atrioventricular valves, and atrial functional TR/MR. Furthermore, a blood access shunt for renal dialysis overloaded the patient’s heart. Although renal failure and disuse syndrome cause systemic failure, leading directly to the patient’s death, surgical intervention before the progression of arrhythmia and/or valvular dysfunction is still recommended. Therefore, it is very important to make a true diagnosis as early as possible.

Optimal timing for surgical intervention is also very relevant. The indications for the operation in case 4 may be controversial because the Qp/Qs was 1.42 according to the catheter analysis, while a Qp/Qs of 2.0 was determined using echocardiography. Some may suggest that it was too early to decide to perform a surgical intervention in case 4. However, the isolated CS ASD was closed because of dyspnea and dilation of the RA and RV. As noted above, in case 5, the blood access shunt for renal dialysis had been overloading the patient’s heart before the operation for 34 years, which may have been an additional and fatal load for this patient specifically. In contrast to the patient in case 5, the patient in case 1 has lived in very good health for 15 years after the operation, even with renal dialysis, which was introduced due to renal sclerosis 10 years after the repair.

It is often difficult to diagnose CS ASD. Notably, the patients in cases 1 and 2 had no preoperative diagnosis of CS ASD. In case 1, because of the large secundum ASD, the echocardiographer might have disregarded other congenital anomalies. Intraoperatively, the initial CP was performed in an anterograde manner from the aortic root, and cannulation for retrograde CP was performed from the CS ostium directly, so blind cannulation injury to the CS roof was impossible. In case 2, preoperative TTE revealed CS enlargement but did not show the defect on the CS roof or the intracardiac shunt. Retrospectively, preoperative computed tomography (CT) was performed only in the axial view, so the defect could have been overlooked. The patient in case 2 had previously undergone RFCA to treat frequent PVCs associated with the RVOT; the catheter was not inserted into the CS, so iatrogenic damage to the roof of the CS caused by the catheter was impossible. TEE, MRI, and three-dimensional CT are quite helpful for diagnosing both CS ASD and other concomitant congenital anomalies [4, 11–13].

In this case series, there were no cases of PLSVC, and each CS roof defect was closed from the left atrial side through a right-side left atrial approach in 2005 and 2013 and a trans-interatrial septal approach in 2016. URCS is a rare congenital heart disease [1, 2], and the syndrome comprises a wide spectrum of anomalies according to the literature [5], ranging from total to partial defects of the CS roof with or without PLSVC and with or without other congenital anomalies. Quaegebeur and colleagues reported that 75% of 24 URCS patients treated over 10 years had PLSVC, in which venous blood is returned correctly into the RA with the roofing technique or baffle rerouting technique. In a study by Quaegebeur and colleagues, these defects were corrected in the LA [5], but they can also be treated with simple ligation following pressure monitoring, as reported by Ootaki and coworkers [3]. Through the left atrial approach, the heart morphology, especially that of the interior LA, can be confirmed to identify partial or total roof defects and examine the PLSVC orifice. Readers may question whether two of the three cases in 2016 required additional AXC to close the residual shunt intraoperatively. The defects were not small, as shown in Figs. 1c and 3, and the surgical view was spatially limited, as shown in Fig. 4. Even with a very experienced adult cardiac surgery team, it is important to obtain a sufficient surgical view and apply appropriate suturing techniques to avoid stenosis of the CS and residual shunting, especially in atypical surgical cases, such as with this anomaly. No disorders of the conduction system, such as atrioventricular block, were encountered after the repair procedure. URCS has atrioventricular septal defect as the most commonly associated major cardiac anomaly [14]. The atrioventricular node is near the orifice of the CS in atrioventricular septal defect cases. Therefore, care should be taken not to place deep stitches near the orifice when suturing the defect of the roof for closure.

Repairs were performed from the left atrial side through standard and orthodox approaches, although simple closure of the CS orifice is also indicated for URCS without PLSVC, with permissible right-to-left shunts [5]. There have been some innovative reports. Takahashi and colleagues reported the anatomical correction of an isolated CS ASD from the right atrial side and closure of the defect through the CS orifice [15]. In addition, Bozso and coworkers reported that through a periareolar approach, an isolated CS ASD was closed successfully via minimally invasive endoscopic repair [16]. Current state-of-the-art endovascular procedures can be indicated for various intracardiac structural diseases. Duarte and colleagues recently reported successful closure of a partially unroofed mid portion of the CS (Kirklin and Barratt-Boyes type III URCS) with a covered stent through PLSVC [17]. An isolated CS ASD was also closed successfully with a percutaneous device closure technique by Sandeep et al. [18] However, the safety and efficacy of these approaches have not yet been established because of limited clinical experience [18]. Therefore, open-heart surgery is often still essential for the repair of CS ASD, as well as ostium secundum ASD without a rigid limbus, sinus venosus ASD, or ostium primum ASD.

There are several limitations to this study. First, this was a retrospective observational study at a single center. Second, over the past 20 years, echocardiography was performed using different equipment in case 1 in 2005 than in the other cases. Third, the surgical approaches were not the same, i.e., the right-side left atrial approach was used in 2005 and 2013, and the interatrial septal approach was used in 2016. Finally, repair was performed from the left atrial side through these two approaches, which was not novel, although standard and orthodox approaches were used. However, the whole interior of the LA could be observed, allowing the type of unroofed CS and other cardiovascular anomalies to be confirmed.

In conclusion, CS ASD was very rare at New Tokyo Hospital from 1999 to 2019. There were five adult patients (Kirklin and Barratt-Boyes type IV URCS) who underwent surgical ASD repair in the past 20 years. One of the patients died approximately 1 year after the operation. Reviewing past reports revealed no patients with a poor prognosis after repair of the same type of anomaly. It is very important to make a true diagnosis as early as possible. When CS enlargement is identified, PLSVC and other anomalies must be ruled out. Otherwise, it can be difficult to identify the true diagnosis. In particular, in cases of an isolated CS ASD, there are often almost no symptoms, and the defect can be missed on TTE [18, 19].

## Supplementary Information

**Additional file 1: Supplemental Table 1.** Number of congenital heart disease and open-heart surgeries performed in the past 20 years. The numbers in parentheses represent the number of concomitant anomalies. The cases were as follows: one case of VSD (type I) with concurrent RVOT stenosis in 2002; one case of secundum ASD with PLSVC in 2003; one case of secundum ASD with sinus venosus ASD and one case of secundum ASD with PDA in 2005; one case of BAV with PDA in 2006; one case of secundum ASD with VSD (type II) and one case of sinus venosus ASD with PAPVR in 2007; one case of sinus venosus ASD with PAPVR and PLSVC in 2008; one case of BAV with PDA in 2009; one case of PDA with CoA in 2010; one case of secundum ASD with PA stenosis in 2012; one case of secundum ASD with PA aneurysm in 2013; one case of BAV with CPAF in 2014; one case of VSD (type II) with a double-chamber RV in 2015; and one case of sinus venosus ASD with concomitant PAPVR in 2017. The details of “others” in Table 6 are as follows: one case of QAV in 1999 and one in 2016; two cases of QAV in 2005; one case of aortopulmonary arterial fistula in 2008; and one case of PA aneurysm in 2013. In the past 20 years, the reoperations were as follows: secundum ASD patch reclosure was performed because of detachment of a previous ASD patch in a 62-year-old woman in 2005, an 81-year-old woman in 2012, and a 45-year-old man in 2015; MVP and TAP were performed because of recurrent MR and TR after atrioventricular septal defect repair was performed in a 68-year-old woman in 2008; mitral valve replacement and MVP were performed because of recurrent MR after primary ASD repair in a 73-year-old man in 2009 and a 70-year-old woman in 2013; and PDA division and closure because of recurrent PDA were performed in a 73-year-old woman in 2010. *ASD* atrial septal defect, *URCS* unroofed coronary sinus, *VSD* ventricular septal defect, *I* type I VSD, *II* type II VSD, *PAPVR* partial anomalous pulmonary venous return, *AVSD* atrioventricular septal defect, *ToF* tetralogy of Fallot, *BAV* bicuspid aortic valve, *CoA* coarctation of the aorta, *PDA* patent ductus arteriosus, *Ebstein* Ebstein anomaly, *CPAF* coronary artery–pulmonary artery fistula, *OHS* open-heart surgery, *PA* pulmonary artery, *QAV* quadricuspid aortic valve.

## Data Availability

The datasets used and/or analyzed during the current study are available from the corresponding author on reasonable request.

## Abbreviations

AF: Atrial fibrillation
AR: Aortic regurgitation
ASD: Atrial septal defect
AXC: Aortic cross-clamping
BAV: Bicuspid aortic valve
CoA: Coarctation of the aorta
CP: Cardioplegia
CPAF: Coronary artery-pulmonary artery fistula
CS: Coronary sinus
CT: Computed tomography
CTR: Cardiothoracic ratio
CXR: Chest X-ray
ECG: Electrocardiography
IVC: Inferior vena cava
LA: Left atrium
MAP: Mitral annuloplasty
MR: Mitral regurgitation
MRI: Magnetic resonance imaging
MVP: Mitral valve repair
NYHA: New York Heart Association
PA: Pulmonary artery
PAPVR: Partial anomalous pulmonary venous return
PDA: Patent ductus arteriosus
PLSVC: Persistent left superior vena cava
ppp: Polypropylene
Pp/Ps: Pulmonary-to-systemic pressure ratio
PVC: Paroxysmal ventricular contraction
QAV: Quadricuspid aortic valve
Qp/Qs: Pulmonary-to-systemic blood flow ratio
RA: Right atrium
RFCA: Radiofrequency catheter ablation
RUPV: Right upper pulmonary vein
RV: Right ventricle
RVOT: Right ventricular outflow tract
SVC: Superior vena cava
TAP: Tricuspid annuloplasty
TEE: Transesophageal echocardiography
TR: Tricuspid regurgitation
TTE: Transthoracic echocardiography
URCS: Unroofed coronary sinus syndrome
VSD: Ventricular septal defect
