# Supplementary material for: Coronary sinus atrial septal defects in adults over the past 20 years at new Tokyo hospital: case series
Source: J Cardiothorac Surg. 2021 May 29;16:150. doi: 10.1186/s13019-021-01522-x (PMC8164258; doi:10.1186/s13019-021-01522-x)
Supplement: Supplementary file 1 — Additional file 1: Supplemental Table 1. Number of congenital heart disease and open-heart surgeries performed in the past 20 years. The numbers in parentheses represent the number of concomitant anomalies. The cases were as follows: one case of VSD (type I) with concurrent RVOT stenosis in 2002; one case of secundum ASD with PLSVC in 2003; one case of secundum ASD with sinus venosus ASD and one case of secundum ASD with PDA in 2005; one case of BAV with PDA in 2006; one case of secundum ASD with VSD (type II) and one case of sinus venosus ASD with PAPVR in 2007; one case of sinus venosus ASD with PAPVR and PLSVC in 2008; one case of BAV with PDA in 2009; one case of PDA with CoA in 2010; one case of secundum ASD with PA stenosis in 2012; one case of secundum ASD with PA aneurysm in 2013; one case of BAV with CPAF in 2014; one case of VSD (type II) with a double-chamber RV in 2015; and one case of sinus venosus ASD with concomitant PAPVR in 2017. The details of “others” in Table 6 are as follows: one case of QAV in 1999 and one in 2016; two cases of QAV in 2005; one case of aortopulmonary arterial fistula in 2008; and one case of PA aneurysm in 2013. In the past 20 years, the reoperations were as follows: secundum ASD patch reclosure was performed because of detachment of a previous ASD patch in a 62-year-old woman in 2005, an 81-year-old woman in 2012, and a 45-year-old man in 2015; MVP and TAP were performed because of recurrent MR and TR after atrioventricular septal defect repair was performed in a 68-year-old woman in 2008; mitral valve replacement and MVP were performed because of recurrent MR after primary ASD repair in a 73-year-old man in 2009 and a 70-year-old woman in 2013; and PDA division and closure because of recurrent PDA were performed in a 73-year-old woman in 2010. ASD atrial septal defect, URCS unroofed coronary sinus, VSD ventricular septal defect, I type I VSD, II type II VSD, PAPVR partial anomalous pulmonary venous return, AVSD atrioventricul [file 13019_2021_1522_MOESM1_ESM.pdf]

Supplemental Table 1 Number of congenital heart disease and open-heart surgeries performed in the past 20 years

	ASD				VSD		PAPVR	AVSD	ToF	BAV	CoA	PDA	Ebstein	CPAF	Others	Congenital total	OHS
	URCS	Secundum	Primary	Sinus venosus	I	II											
1999		4	1							2					1	8	348
2000		4								3						7	366
2001		10				1				7						18	353
2002		7			1	1				6					(1)	15	355
2003		8	1			2				9					(1)	20	394
2004		6	1							5						12	309
2005	1	11 (+1)		(1)	1					6		(1)			2	21	269
2006		7			1	1			1	4	1	1 (+1)				16	285
2007		9		1		1 (+1)	(1)		1	4		1				17	288

2008		10	1	1	1		(1)	1	5		1		1	1 (+1)	22	352
2009		3				2			10		1 (+1)		1		17	407
2010		4				2			4	(1)	2	1	1		14	403
2011		6			1	1			9		1	1	3		22	396
2012		6				3			16	1	2		1	(1)	29	427
2013	1	10	1			2			13		1		1	1 (+1)	30	418
2014		2			1				17				(1)		20	379
2015		4				2			9				1	(1)	16	299
2016	3	1				1			12				1	1	19	260
2017		1		1			(1)		11				1		14	300
2018		2	1			2	1		11		1		1		19	350
2019			1			3		1	10						15	356

Total	5	115 (+1)	7	3 (+1)	6	24 (+1)	1 (+3)	2	2	173	2 (+1)	11 (+3)	2	12 (+1)	6 (+6)	371	7314
